# Non-coding RNAs in Host–Pathogen Interactions: Subversion of Mammalian Cell Functions by Protozoan Parasites

**DOI:** 10.3389/fmicb.2017.00474

**Published:** 2017-03-21

**Authors:** Ethel Bayer-Santos, Marjorie M. Marini, José F. da Silveira

**Affiliations:** ^1^Departamento de Bioquímica, Instituto de Química, Universidade de São PauloSão Paulo, Brazil; ^2^Departamento de Microbiologia, Imunologia e Parasitologia, Escola Paulista de Medicina, Universidade Federal de São PauloSão Paulo, Brazil

**Keywords:** non-coding RNA, miRNA, extracellular vesicles, protozoan parasites, parasitic diseases, infection

## Abstract

Pathogens have evolved mechanisms to modulate host cell functions and avoid recognition and destruction by the host damage response. For many years, researchers have focused on proteins as the main effectors used by pathogens to hijack host cell pathways, but only recently with the development of deep RNA sequencing these molecules were brought to light as key players in infectious diseases. Protozoan parasites such as those from the genera *Plasmodium*, *Toxoplasma*, *Leishmania*, and *Trypanosoma* cause life-threatening diseases and are responsible for 1000s of deaths worldwide every year. Some of these parasites replicate intracellularly when infecting mammalian hosts, whereas others can survive and replicate extracellularly in the bloodstream. Each of these parasites uses specific evasion mechanisms to avoid being killed by the host defense system. An increasing number of studies have shown that these pathogens can transfer non-coding RNA molecules to the host cells to modulate their functions. This transference usually happens via extracellular vesicles, which are small membrane vesicles secreted by the microorganism. In this mini-review we will combine published work regarding several protozoan parasites that were shown to use non-coding RNAs in inter-kingdom communication and briefly discuss future perspectives in the field.

## Introduction

Protozoan parasites comprise an exceptionally diverse group of unicellular eukaryotic organisms. Some of them are etiological agents of human parasitic diseases leading to significant morbidity and mortality, with high social and economic impacts, particularly in the tropics. In addition to medical and veterinary importance, protozoan parasites are interesting models for studying the evolution of eukaryotic cells, since they diverged early in the evolution of eukaryotes.

Protozoan parasite-host cell interplay can occur directly by physical cell-cell contact or indirectly via secreted/excreted molecules, which can be released into the extracellular medium or packed into extracellular vesicles (EVs) (reviewed in Torrecilhas et al., 2012; Barteneva et al., 2013; Deolindo et al., 2013; Mantel and Marti, 2014; Marcilla et al., 2014; Judice et al., 2016; Marti and Johnson, 2016; Roditi, 2016; Szempruch et al., 2016a; Watanabe Costa et al., 2016). The packaging of proteins and nucleic acids, such as small non-coding RNAs (sncRNAs), in EVs protects them from extracellular degradation until they reach the target recipient cell. Transference of proteins and RNAs within EVs seems to be a safe mechanism for local and systemic intercellular interactions between parasites and host cells, and also between parasites themselves. The exchange of information may take place in both directions, from parasite to host or vice-versa, and may be beneficial or detrimental to the parasite. We herein present the current understanding of sncRNAs in protozoan parasites and provide examples of their role during infections.

### Biogenesis of sncRNAs in Human Protozoan Parasites

Small non-coding RNAs range in size from 20 to 300 nucleotides and comprise a large variety of small RNAs, including small interfering RNA (siRNA), microRNA (miRNA), transfer RNA (tRNA), 5S ribosomal RNA (5S rRNA), small nuclear RNA (snRNA), small nucleolar RNA (snoRNA) and 7SL cytoplasmic RNA (7SL RNA). Protozoan parasites show a variety of RNA interference (RNAi) pathways and complex miRNA repertoires, which differ from those in mammalian cells in several features, like the organization and structure of the main components of the RNA-induced silencing complex (RISC) and the nature of the precursor RNA, which could be derived from snoRNA, tRNA, rRNA, satellite DNA, natural antisense transcripts (NATs), or transcripts from transposons and retrotransposons.

Protozoan miRNAs do not share significant homology to known miRNAs of plants and animals. Dicer, Argonaute and Piwi genes (essential components of RISC) of protozoan parasites are grouped in a distinct phylogenetic lineage from metazoan (Braun et al., 2010; Batista and Marques, 2011; Zheng et al., 2013; Garcia-Silva et al., 2014c). In addition, protozoa lack Drosha (a nuclear RNase III) homologs, suggesting that a Drosha-independent pathway is present in these parasites. RNA-dependent RNA polymerase like-genes, which encode an enzyme implicated in the amplification of pre-miRNAs, have been identified in *Giardia lamblia, Entamoeba histolytica*, and *Toxoplasma gondii.* The main RISC components (Argonaute, Piwi, Dicer, RNAse III) and miRNAs have been identified in *Trypanosoma brucei, Trypanosoma congolense, Leishmania* (*V.*) *braziliensis, T. gondii, Neospora caninum, G. lamblia, Trichomonas vaginalis* and *E. histolytica*, suggesting that these organisms have a classical RNAi pathway (Militello et al., 2008; Atayde et al., 2011, 2013; Hakimi and Cannella, 2011; Kolev et al., 2011; Zheng et al., 2013).

Bioinformatic and functional analyses showed that *Trypanosoma rangeli, Leishmania donovani, Leishmania major, Trypanosoma cruzi, and Plasmodium falciparum* are RNAi-deficient organisms (Robinson and Beverley, 2003; DaRocha et al., 2004; Baum et al., 2009; Atayde et al., 2011; Zheng et al., 2013; Stoco et al., 2014). Orthologs of RNAi machinery components are present only as pseudogenes in *T. rangeli, L. major, Leishmania infantum*, and *Leishmania mexicana* (Zheng et al., 2013; Stoco et al., 2014). Although, *T. cruzi* is not able to respond to dsRNA (DaRocha et al., 2004), it expresses an Argonaute/Piwi protein (Garcia Silva et al., 2010b, Garcia-Silva et al., 2014c). It has been suggested that some sncRNAs that have been conserved during evolution, such as snoRNA and tRNA, could belong to the most primitive small RNA pathways from which the canonical RNA silencing pathways have evolved (Garcia-Silva et al., 2012; Zheng et al., 2013). It is worth highlighting that a failure to identify protein homologs using bioinformatics in genome databases does not imply that the protein function was in fact lost. In these cases, it is possible that primary sequences diverged too much to be detected by sequence homology.

The hypothesis that RNAi-deficient parasitic protozoa may have non-classical RNAi pathways is very attractive. However, we cannot rule out the possibility that these organisms may have regulatory genetic mechanisms mediated by other RNAs than small interfering RNAs. For instance, long non-coding RNAs (lncRNAs) which can be transcribed as whole or partial NATs to coding genes. NATs have been detected in many protozoan parasites such as *P. falciparum, T. gondii*, *T. parvum*, *T. brucei*, *Leishmania* spp., and *G. lamblia* (Militello et al., 2008). Finally, high-throughput sequencing of RNAs bound to miRNP (micro RNA ribonucleoproteins) and other RNPs (small nuclear RNP, heterogeneous nuclear RNP, cytoplasmic RNP) would be necessary to determine the full RNA repertoire of these protozoa. These studies may reveal new classes of non-coding regulatory RNAs.

## Interplay Between Protozoan Parasites, Mammalian Cells And Vectors: Possible Roles For sncRnas

Descriptions of non-coding RNAs in host–protozoan parasites interactions are discussed below and a summary of references with main findings is shown in **Table 1**.

**Table 1:** Examples of non-coding RNAs in host–protozoan parasites interactions.

Protozoan	Host	Experimental evidences	Reference
*Trypanosoma cruzi*	Human	*T. cruzi* extracellular vesicles (EVs) deliver sncRNA cargo into HeLa cells conferring susceptibility to infection and changing expression of genes related to cytoskeleton, extracellular matrix, and immune responses pathways	Bayer-Santos et al., 2013, 2014; Garcia-Silva et al., 2014a,b; Fernandez-Calero et al., 2015
	Human and murine	Dysregulation of miRNAs of myocardial tissue in human chronic Chagasic cardiomyopathy (CCC) and in murine *T. cruzi* acute infection Overexpression of lncRNA-myocardial infarction-associated transcript (MIAT) in human CCC and murine *T. cruzi* acute infection	Ferreira et al., 2014; Navarro et al., 2015; Frade et al., 2016
	Murine	Up-regulation of miRNAs of thymic epithelial cells in *T. cruzi*-induced thymic atrophy	Linhares-Lacerda et al., 2015
*Leishmania* spp.	Murine and human	Dysregulation of miRNA expression in *L. major*-infected murine and human primary macrophages. Up-regulation of miRNAs targeting MAP kinase, JAK-STAT and TGFβ signaling pathways in human monocyte derived dendritic cells and macrophages	Lemaire et al., 2013; Frank et al., 2015; Geraci et al., 2015
	Murine	Extracellular vesicles secreted by *L. donovani* downregulated miR-122 activity in hepatic cells. *Leishmania* metalloprotease gp63 targets pre-miRNA processor Dicer1 to prevent miRNP formation in hepatic cells	Ghosh et al., 2013
	Human	*L. donovani* and *L. braziliensis* EVs deliver specific sncRNAs into human macrophages	Lambertz et al., 2015
	Human	Autophagic machinery of bone marrow-derived macrophages (BMDM) is activated in *L. major* infection. Transfection of BMDM with specific siRNAs against autophagy-related genes or inhibitors of autophagy-associated miRNAs inhibited autophagic digestion of *L. major*	Singh et al., 2016
	Murine and human	Downregulation of vacuolar sorting protein HRS in *L. donovani*-infected macrophages prevents uncoupling of mRNA-AGO2 interaction, blocking degradation of translationally repressed messages. let-7a miRNPs fail to repress newly formed IL-6 mRNA. Translation of IL-6 helps *Leishmania* to suppress host macrophage activation and promote infection	Bose et al., 2017
*Plasmodium* ssp.	*Anopheles gambiae*	Depletion of Argonaute I and Dicer I in the mosquito *A. gambiae* during *P. berghei* infection led to a two-fold increase in the number of oocysts	Winter et al., 2007
	Human	In the *Plasmodium* intraerythrocytic cycle, miRNAs (let-7i and miR-451) are transferred from sickle cell erythrocytes to the *P. falciparum* inhibiting the parasite growth	LaMonte et al., 2012
	Human and murine	*P. falciparum*-infected erythrocyte releases EVs carrying functional miRNAs, which are internalized by endothelial cells altering gene expression and barrier properties in endothelial cells	Mantel et al., 2016
	Human and murine	Blocking miR-155 function in an experimental mouse model of cerebral malaria by gene knockout or pre-treatment with miR-155 antagomir enhances endothelial quiescence, blood-brain-barrier integrity and host survival	Barker et al., 2017
*Toxoplasma gondii*	Human and murine	Alteration of miRNA profiles in *T. gondii*-infected human fibroblasts. miR-146a and miR-155, involved in response to *T. gondii* challenge, were induced in mouse brain during *T. gondii* infection	Zeiner et al., 2010; Cannella et al., 2014
*Cryptosporidium parvum*	Human	*C. parvum* infection dysregulates miRNA expression in human cholangiocytes. Downregulation of some miRNAs (e.g., let-7 miRNA and miR-221) increases infiltration of lymphocytes into the intestinal mucosa and reinforces epithelial defense response against *C. parvum*. Inhibition of other miRNAs (mir-125b-1, mir-21, mir-30b, and mir-23b-27b-24-1 cluster genes) increases *C. parvum* burden	Chen et al., 2007; Zhou et al., 2009; Gong et al., 2011
*Trichomonas vaginalis*	Human	*T. vaginalis* trophozoites release exosomes carrying small RNAs that fuse with human ectocervical cells to deliver their cargo. Exosomes modulate secretion of proinflammatory cytokines IL-6 and IL-8, which are regulated by endogenous miRNAs	Twu et al., 2013


### Intracellular Protozoan Pathogens

Invasion and replication inside a mammalian cell is a common strategy used by protozoan pathogens to evade the host immune system. Protozoan parasites may modulate the expression of host miRNAs associated with different biological processes in order to survive in the intracellular environment. Conversely, host miRNAs can inhibit the proliferation of microorganisms by targeting virulence and essential genes of the parasite (Asgari, 2011).

*Plasmodium* spp., *T. gondii*, and *Cryptosporidium parvum* are medically important protozoa that belong to the Apicomplexa phylum. As obligate intracellular parasites, they use different mechanisms to reorganize host cell functions to allow their survival and replication inside host cells (Plattner and Soldati-Favre, 2008). Apicomplexans were reported to change the host cell miRNA expression profile. Changes in miRNA expression might be induced by the parasite or be a defensive response from the host cell (Hakimi and Cannella, 2011). Although it was reported that members of let-7 family were downregulated during infection by different apicomplexan parasites, most changes detected in miRNA expression profile are species-specific. miR-155, which regulates the development and function of immune cells, is upregulated in *T. gondii* infection and downregulated in *Cryptosporidium* infection (Lindsay, 2008; Zhou et al., 2009; Zeiner et al., 2010). There is a decrease in expression of miRNAs let-7 and mir-221 in epithelial cells infected with *C. parvum*, leading to upregulation of ICAM-1 (intercellular adhesion molecule-1) (Chen et al., 2007). Expression of ICAM-1 allows the adhesion and recognition of immune cells, modulating the response against infection (Gong et al., 2011). Another example of host cell miRNome (the full spectrum of miRNAs expressed in a specific genome) alterations was observed in *T. gondii*-infected fibroblasts in which the expression of about 14% of miRNAs was affected (Zeiner et al., 2010). Different studies showed changes in a subset of miRNAs, including miR-146a and miR-155, which are important in host cell responses to *T. gondii* infection (Vigorito et al., 2013; Cannella et al., 2014; Saba et al., 2014).

**FIGURE 1:**
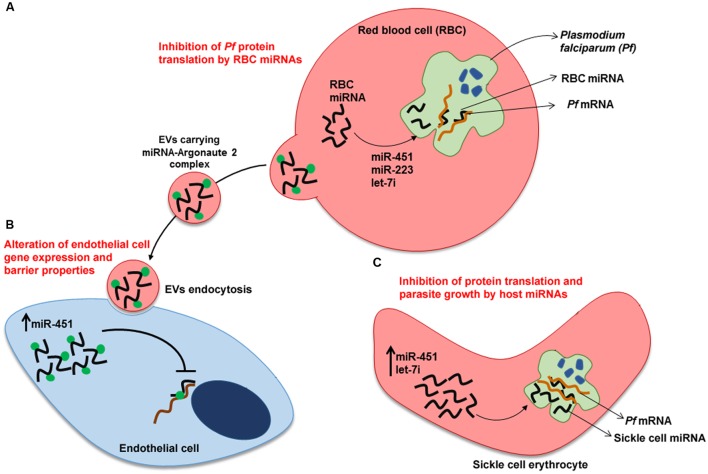
**Human miRNAs response to *Plasmodium falciparum* infection.** **(A)** *Plasmodium*-infected red blood cells (RBC). Translocation of miRNAs from RBC to *P. falciparum* inhibit translation of specific mRNAs of the parasite. **(B)** Transfer of miRNAs from *Plasmodium*-infected RBC to endothelial cells. Endocytosis by endothelial cell of EVs released by *Plasmodium*-infected RBC. miRNA-Argonaute 2 complexes alter gene expression and barrier properties in endothelial cells, contributing to malaria resistance. **(C)** *Plasmodium*-infected sickle cell erythrocyte. Translation inhibition of parasite mRNAs by sickle cell erythrocyte-miRNAs. miR-223, miR-451and let-7i inhibit parasite growth. Upward arrows denote an increase in expression of miR-451 and let-7i. EV, extracellular vesicle.

Sickle cell erythrocytes show hemoglobin polymorphism (HbS) in one or both alleles resulting in red blood cells with a sickle shape. Populations living in malaria endemic areas have a high frequency of this gene probably as a result of its protective effect during *Plasmodium* infection (Aidoo et al., 2002). LaMonte et al. (2012) elegantly showed that about 100 human miRNAs are transferred to the *P. falciparum* during the intraerythrocytic cycle (**Figure 1**). Two miRNAs (let-7i and miR-451) that negatively modulate parasite growth were shown to be enriched in sickle cell erythrocytes. These miRNAs bind to specific *Plasmodium* mRNAs, inhibiting translation by blockage of ribosomal loading. Thus, resistance of sickle cell erythrocytes to malaria infection may be in part due to a protective dysregulated miRNA expression in these cells.

Erythrocyte miRNAs also play an important role in communication between host cells during *Plasmodium* infection. Infected erythrocyte releases EVs carrying functional miRNAs (Argonaute 2 complexes, enriched with miR-451 and let-7b) in the bloodstream (**Figure 1**). These EVs are internalized by endothelial cells and the delivered miRNAs change recipient cell gene expression, culminating in altered endothelial cell barrier properties (Mantel et al., 2016).

miR-155 seems to have an important role in the pathogenesis of cerebral malaria via negative regulation of blood-brain-barrier integrity and T cell function. In a mouse experimental model of cerebral malaria, Barker et al. (2017) demonstrated that blocking miR-155 function by gene knockout or pre-treatment with miR-155 antagomir enhances endothelial quiescence, blood-brain-barrier integrity and host survival. Further investigation is needed to explore the potential therapeutic use of miR-155 in the treatment of cerebral malaria.

Another example of the importance of miRNA in the communication between host–parasite can be observed between the insect vector *Anopheles gambiae* and *Plasmodium*. Winter et al. (2007) identified differences in expression levels of four miRNA in the midgut of *A. gambiae* infected with *Plasmodium*. Depletion of Argonaute I and Dicer I of the insect vector during *Plasmodium* infection led to a two-fold increase in the number of oocysts in the mosquito. It was suggested that these enzymes play an important role in the mosquito’s resistance to *P. berghei* infection, probably due to post-transcriptional regulation mediated by miRNA genes involved in defense mechanisms.

The trypanosomatids *T. cruzi* and *Leishmania* spp. are transmitted by insect vectors to mammalian hosts. *L. braziliensis* has been reported to have a functional siRNA pathway, while *T. cruzi*, *L. major*, and *L. amazonensis* do not have a functional RNAi machinery (Atayde et al., 2011; Kolev et al., 2011). However, *T. cruzi* expresses an AGO/PIWI protein (TcPIWI-tryp) that colocalizes with tRNA-derived small RNAs (stRNA) in specific cytoplasmic granules which could represent secretory organelles (Garcia-Silva et al., 2010a). It is known that *T. cruzi* releases different populations of EVs (Bayer-Santos et al., 2013) that contain different sets of small RNAs, including stRNAs (Bayer-Santos et al., 2014; Garcia-Silva et al., 2014a; Fernandez-Calero et al., 2015). EVs containing stRNAs and TcPIWI-tryp were shown to be transferred between *T. cruzi* cells, increasing their differentiation rate, and between parasites and mammalian host cells, inducing changes in gene expression (Garcia-Silva et al., 2014b).

Inflammatory lesions not directly related to the presence of *T. cruzi* have been reported in patients with chronic Chagas disease cardiomyophaty (CCC) and in a murine model (Pinho et al., 2002). Treatment of mice with *T. cruzi* EVs before the infection induced severe heart pathology and inflammation, and higher levels of proinflammatory cytokines and nitric oxide, suggesting that EVs are involved in the pathology (Torrecilhas et al., 2012; Nogueira et al., 2015). miRNAs were significantly altered in CCC as compared to idiopathic dilated cardiomyopathy, suggesting that miRNA may regulate gene expression in CCC pathogenesis (Ferreira et al., 2014; Navarro et al., 2015; Linhares-Lacerda and Morrot, 2016). The long ncRNA MIAT (myocardial infarction-associated transcript) was overexpressed in patients with CCC and in mice, indicating that MIAT could be a specific biomarker of CCC (Frade et al., 2016). An additional work showed that *T. cruzi* infection of thymic epithelial cells induce changes in miRNAs levels of host cells (Linhares-Lacerda et al., 2015; Linhares-Lacerda and Morrot, 2016). It was also shown that mammalian cells incubated with *T. cruzi* can release EVs which bound to trypomastigotes and protect them against lysis by the complement system (Cestari et al., 2012; Ramirez et al., 2017).

*Leishmania donovani* and *L. braziliensis* were also reported to release EVs containing RNAs (Lambertz et al., 2015). In agreement with data from *T. cruzi* EVs, sequencing *L. donovani and L. braziliensis* exosomal RNA indicated that the majority of cargo sequences were derived from sncRNA species such as rRNA and tRNA, suggesting that the packaging of specific RNA sequences into EVs may be a conserved phenomenon (Lambertz et al., 2015). In *L. braziliensis*, which is an RNAi-competent organism, it was also found sequences derived from siRNA-coding regions in both sense and anti-sense, suggesting that these molecules might be packaged in EVs as double-stranded RNAs (Lambertz et al., 2015). Additional work showed that infection of macrophages (Lemaire et al., 2013; Frank et al., 2015; Geraci et al., 2015) and dendritic cells (Geraci et al., 2015) by *Leishmania* changes host cell miRNA expression profile. However, the mechanism regulating such changes were not determined. Conversely, mechanistic insight was given in regard of the *Leishmania*-induced reduction of miR-122 in host cells. Delivery of Zn-metalloprotease surface glycoprotein GP63 via EVs to mammalian host cells, allow GP63 to target pre-miRNA processor Dicer1 and prevent miRNP formation, resulting in reduction of miR-122 activity and an increase in parasite burden (Ghosh et al., 2013).

Recently, Bose et al. (2017) proposed a new mechanism to explain the dysregulation of host cell miRNA expression by *L. donovani*. It involves the blocking of maturation of endosomes to late endosome and MVBs by targeting the endosomal protein HRS (hepatocyte growth factor regulated tyrosine kinase substrate), a vacuolar protein sorting (VPS) protein. Downregulation of HRS in *L. donovani*-infected macrophages prevents uncoupling of mRNA-AGO2 interaction, blocking degradation of translationally repressed messages and recycling of miRNPs. let-7a miRNPs remain bound to target mRNAs and fail to repress IL-6 mRNA. Enhanced translation of IL-6 in host cells helps *Leishmania* to suppress host macrophage activation and promote infection.

Autophagic machinery of bone marrow-derived macrophages (BMDMs) is activated in *L. major* infection. Singh et al. (2016) have provided mechanistic details on the regulation of this process by host miRNAs. Expression of MIR30A-3p is enhanced during *L. donovani* infection, suggesting a regulatory role of this miRNA in the modulation of host cell autophagy. Transfection of BMDM with specific siRNAs against autophagy-related genes or inhibitors of autophagy-associated miRNAs inhibited autophagic digestion of *L. major*. BECN1/Beclin 1, a key autophagy-promoting protein, is a potential target of MIR30A-3p which negatively regulates BECN1 expression. The regulation of *L. donovani* autophagy by targeting BECN1 may have impact on the treatment of visceral leishmaniasis.

### Extracellular Protozoan Parasites

Interaction between extracellular protozoan parasites and host cells is critical for tissue adhesion, colonization and damage. *E. histolytica* and *T. vaginalis* are extracellular parasites that live in the gastrointestinal and urogenital tracts, respectively. These parasites must adhere to epithelial cells to establish a focus of infection. *T. vaginalis* trophozoites express adherence factors that allow attachment to ectocervical epithelial cells. Twu et al. (2013) showed that trophozoites release exosomes carrying small RNAs and parasite-specific proteins that fuse with host cells to deliver their cargo. EVs released from a highly adherent strain of *T. vaginalis*, with tissue-tropic adherence to ectocervical cells, are able to enhance attachment of a poorly adherent strain to both vaginal and prostate epithelial cells. *T. vaginalis* exosomes also modulate the secretion of proinflammatory cytokines IL-6 and IL-8, possibly allowing the parasite to sustain a chronic infection (Twu et al., 2013). Providing that the production of IL-6 and IL-8 is regulated by endogenous miRNAs, it is possible that host miRNA dysregulation control the expression of these cytokines during *T. vaginalis* infection.

Modulation of host cells miRNA profile by the pathogen also occurs in amebic infections. *E. histolytica* kills intestinal epithelial cells after attachment of trophozoite to host cell membranes, leading to a Ca^2+^-induced signaling that culminates in cell death. This is followed by a potent inflammatory response and invasion of colonic crypts by trophozoites (Ralston and Petri, 2011). Whole genome-scale RNAi screening has proved useful for identifying human host factors involved in cell invasion and intracellular growth of *T. cruzi* or host factors required for amebic cytotoxicity (Genovesio et al., 2011; Caradonna et al., 2013; Marie et al., 2015). Marie et al. (2015) used a genome-wide miRNA library to screen human epithelial cells susceptible to killing by *E. histolytica*. After successive rounds of selection with trophozoites, they identified resistant cells to the parasite, showing that RNAi library had increased resistance to *E. histolytica* cytotoxicity when compared to the control library.

*Trypanosoma brucei* and related subspecies are transmitted between mammalian hosts by an insect vector (tsetse fly). In both hosts, they are extracellular and migration to specific host tissues is essential for parasite development and pathogenesis (Ralston et al., 2009). Bloodstream trypomastigotes can move out of blood vessels and invade extravascular tissues, including the central nervous system. In tsetse fly, epimastigotes attach to epithelial cells in the salivary gland and differentiate into metacyclic trypomastigotes, which are infective for humans. Recently, *T. brucei* was reported to produce EVs that are mainly originated from membrane nanotubes (Szempruch et al., 2016b). EVs produced by *T. brucei rhodesiense* contain the serum resistance-associated protein (SRA), which is necessary for human infectivity. *T. brucei rhodesiense* can transfer SRA to non-human infectious trypanosomes via EVs, allowing these parasites to evade the human innate immunity. In addition, *T. brucei* EVs can also fuse with mammalian erythrocytes, altering the physical properties of the membrane and causing erythrocyte lysis and anemia (Szempruch et al., 2016b). Despite this interesting work, there is no report of transference of non-coding RNAs in *T. brucei* to date.

## Concluding Remarks

There is growing number of evidence showing that ncRNAs actively change the outcome of infections. Host miRNA dysregulation has been associated with impaired immune response and increased host colonization by the pathogen. Conversely, miRNAs can be employed by the host as a mechanism of defense against the parasite. EVs seem to intermediate this communication, but in most cases it is not known precisely which molecules are involved. Future investigation is required to identify all ncRNAs molecules and the possible mechanism by which they can be transferred to mammalian cells in different conditions. Likewise, the role of human host-derived EVs on infection is another important field to be explored. Besides its potential as diagnostic and prognostic tools, miRNAs and other ncRNAs are potential targets for chemo- and immunotherapies for parasitic diseases.

## Author Contributions

All authors (EB-S, MM, JdS) conceived and wrote the manuscript. They have made substantial, direct and intellectual contribution to the work, and approved it for publication.

## Conflict of Interest Statement

The authors declare that the research was conducted in the absence of any commercial or financial relationships that could be construed as a potential conflict of interest.
